# Integrated multi-omics characterization of neuroblastoma with bone or bone marrow metastasis

**DOI:** 10.1016/j.gendis.2024.101511

**Published:** 2025-01-03

**Authors:** Kai Huang, Linyu Yang, Yue Ma, Lijian Cao, Suwen Li, Zhenzhen Zhao, Jianwu Zhou, Shan Wang

**Affiliations:** Department of Pediatric Surgical Oncology, The Children's Hospital of Chongqing Medical University, National Clinical Research Center for Child Health and Disorders, Ministry of Education Key Laboratory of Child Development and Disorders, Chongqing Key Laboratory of Pediatric Metabolism and Inflammatory Diseases, Chongqing 400014, China

**Keywords:** Bone marrow metastasis, Bone metastasis, Immunotherapy, Multi-omics, Neuroblastoma, Transketolase (TKT)

## Abstract

The pathogenesis of neuroblastoma with bone or bone marrow metastasis (NB-BBM) and its complex immune microenvironment remain poorly elucidated, hampering the advancement of effective risk prediction for BBM and limiting therapeutic strategies. Feature recognition of 142 paraffin-embedded hematoxylin-eosin-stained tumor section images was conducted using a Swin-Transformer for pathological histology to predict NB-BBM occurrence. Single-cell transcriptomics identified a tumor cell subpopulation (NB3) and two tumor-associated macrophage (TAM) subpopulations (SPP1^+^ TAMs and IGHM^+^ TAMs) closely associated with BBM and highlighted transketolase (TKT) as a key molecular marker for metastatic progression in NB. This extensive multi-omics investigation into NB-BBM enhances our understanding of single-cell transcriptional dynamics in NB beyond existing research, outlining the evolution from *in situ* carcinoma through tumorigenesis to bone marrow metastases. Furthermore, exploration of the immune microenvironment identified specific subpopulations of TAMs crucial in promoting NB-BBM, presenting new avenues for immunotherapy. These insights enhance our understanding of the metastatic process from NB to BBM and facilitate the development of more effective diagnostic and therapeutic strategies for this aggressive pediatric cancer.

## Introduction

Bone and bone marrow represent common sites for tumor metastases, with the occurrence of bone or bone marrow metastases (BBM) often associated with poor prognosis.^1^ Consequently, addressing BBM is crucial in the treatment and management of cancer patients. Neuroblastoma (NB), the most prevalent extracranial solid tumor among children, is characterized by a high rate of metastasis, particularly in patients classified as high-risk, who have an event-free survival rate below 80%.^2^^,^^3^ Furthermore, patients older than 18 months with MYCN oncogene amplification frequently present with hematogenous spread at diagnosis, with incidences of bone marrow and bone metastases reaching as high as 71% and 56%, respectively.^4^ This highlights the critical need to enhance early diagnostic prediction of NB-BBM, innovate novel therapeutic strategies, and elucidate the mechanisms and principal targets associated with the rapid progression of NB and onset of BBM.

Recent advancements in artificial intelligence, especially in deep learning applied to extensive medical datasets, have shown promise in improving cancer diagnosis and treatment modalities in various oncological fields.^5^^,^^6^ In NB, the integration of radiomics with deep learning has surpassed the diagnostic accuracy of traditional imaging technologies.^7^ Furthermore, deep learning for automatic tumor segmentation, using computed tomography-based radiomics, has been successful in predicting MYCN gene amplification status.^8^ Nonetheless, the use of machine learning in conjunction with histopathological analysis for predicting NB-BBM is still limited.

Additionally, the specific genomic landscape of NB-BBM has not yet been characterized, with current research focusing on telomere maintenance mechanisms and genetic and clonal evolution during metastatic progression.^9^^,^^10^ Tumor metastasis embodies a multifaceted and dynamic process, encompassing genetic and epigenetic changes, cellular interactions, and microenvironmental shifts.^11^^,^^12^ To date, single-cell analyses have largely been confined to comparing various primary NB grades^13^, ^14^, ^15^, ^16^ or differentiating between bone marrow-negative or -positive cases,^17^^,^^18^ with some investigations exploring tumor origins.^15^ In the current study, we systematically explored the heterogeneity inherent in disease progression from primary lesions (BBM or non-metastatic) to bone marrow metastases, identifying differential targets across distinct stages of progression. Based on the analysis of diversity in the development of BBM within NB, our study facilitates early prediction of BBM status in NB using pathological images, providing a foundation for advancing future diagnostic and treatment approaches.

## Materials and methods

### Patient cohort and sample collection

This study included a cohort of 167 NB patients from the Children's Hospital of Chongqing Medical University (CH-CMU). The collection comprised 142 paraffin-embedded sections stained with hematoxylin and eosin and single-cell transcriptome sequencing data for 10 patients. This cohort, derived from a retrospective study, included NB patients who received standard treatment protocols at the CH-CMU Department of Surgical Oncology from January 1, 2016, to December 31, 2023. The patients who aged under 18 years, received a primary pathological diagnosis of NB following surgical resection, and had mostly complete clinical information were included. Additionally, this study incorporated sequencing data from 21 single-cell transcriptomes available in the Gene Expression Omnibus (GEO) public database. Specifically, data from 9 cases were obtained from the study GSE137804 (accessible at https://www.ncbi.nlm.nih.gov/geo/query/acc.cgi?acc=GSE137804), and data from 12 cases were sourced from GSE216155 (available at https://www.ncbi.nlm.nih.gov/geo/query/acc.cgi?acc=GSE216155), enabling a comprehensive joint analysis.

### Data preprocessing

Pathological slides of NB patients reviewed by a pathologist were digitized using the slide scanning system SQS-40P (TEKSQRAY, Shenzhen, China), and whole slide images were stored in SVS format. The computer equipment used to perform the experiments was a Raytheon Black Warrior computer equipped with an NVIDIA GeForce RTX 4070 graphics card. The 142 examples of images collected in this study were randomly divided into training, validation, and test sets in the ratio of 8:1:1. Due to the large size of the images, image slice extraction is a common preprocessing step in deep learning, especially when dealing with larger images or when the image needs to be segmented into smaller pieces for processing. This study used the Openslide toolkit to read the slices and extract the sliced region as a PNG image with an image size of 1024 ∗ 1024 pixels. After the images were collected, the color of the images varied due to their staining, so the color of the images needed to be normalized. This study used the OpenCV library to convert all the images into greyscale maps, reducing the differences between the cases before and after the image conversion, as shown in Figure S1A.

### Feature extraction by Swin-Transformer model

Transformer is a deep learning model architecture based on an attention mechanism. At the heart of the Transformer model is the Self-Attention mechanism, through which the model can establish global dependencies in a sequence, enabling the representation of each position to take into account the information of other positions. The Swin-Transformer model was employed as a feature extractor for all the sliced images in this work. The Swin-Transformer model used in this study took the extracted image of size 1024 ∗ 1024 pixels and after network computation, it became a feature tensor of size 1534 ∗ 768 pixels.

### Single-cell data analysis

Ten patient single-cell samples were obtained from the CH-CMU Department of Surgical Oncology; in addition, data on these single-cell cases were obtained from previous studies. These samples were transformed into single-cell suspensions and loaded onto a microfluidic device, then processed to construct single-cell RNA sequencing libraries using a GEXSCOPE single-cell RNA library kit (Singleron Biotechnologies, China) following the manufacturer's protocols. The resultant dataset was integrated with two GEO datasets for comprehensive analysis. The Seurat R package was used for standard downstream processing of the single-cell sequencing data. Dimensionality reduction and cluster analysis were performed to identify distinct cellular populations, with an emphasis on tumor cells and macrophages.

Cells were categorized into 10 clusters based on gene expression patterns, representing different subpopulations of NB cells and macrophages. These included cluster 1 NB0 (expressing GNB2L1), cluster 2 NB1 (expressing CHGB), cluster 3 NB2 (expressing HIST1H4C and TYMS), cluster 4 NB3 (expressing UBE2C and TOP2A), cluster 5 FCN1^+^ tumor-associated macrophages (TAMs; expressing FCN1 and PLAC8), cluster 6 APOD^+^ TAMs (expressing APOD and GPNMB), cluster 7 SPP1^+^ TAMs (expressing SPP1), cluster 8 IGHM^+^ TAMs (expressing IGHM and MS4A1), cluster 9 CCL5^+^ TAMs (expressing CCL5 and CCL7), and cluster 10 STMN2^+^ TAMs (expressing STMN2 and DDX1). The InferCNV package was used to evaluate the copy number variations in primary tumors, comparing metastatic (G2), non-metastatic (G1), and BM (G3) subgroups. Functional enrichment analysis of Kyoto Encyclopedia of Genes and Genomes (KEGG) pathways was performed using gene set variation analysis (GSVA) on the identified NB3 cell subpopulations in subgroups G1, G2, and G3.

For the developmental trajectory construction of TMAs, pseudo-time analysis was conducted using the “Monocle” package (v2.28.0), revealing the progression of gene expression over time. Cell–cell communication was analyzed using CellChat, focusing on receptor–ligand interactions to infer signaling pathways and regulatory networks within and between cell subtypes. The evaluation of ligand-receptor gene interactions between different cell types was conducted by merging the count of these interactions based on the L2 paradigm with the activity level of downstream transcription factors, as calculated using the integrated gene set enrichment analysis (GSEA) algorithm.

### Real-time quantitative PCR

Total RNA was extracted using TRIzol reagent (Invitrogen) following the manufacturer's recommendations. Subsequently, cDNA was synthesized from 200 ng of RNA using an Evo M-MLV RT Mix Kit (Accurate Biology, China). Real-time quantitative PCR was performed using a SYBR Green Premix Pro Taq HS qPCR Kit II (Accurate Biology). All primers (Beijing Tsingke Biotech Co., Ltd., China) are listed in Table S1. The relative expression of target genes to ACTIN was calculated using the 2^−DDCT^ method.

### Cell culture and transfection

The SK-N-SH and SH-SY5Y NB cell lines were purchased from Pricella Biotechnology (Wuhan, China). All cells were incubated in a culture medium containing 10% serum and 1% penicillin/streptomycin at 37 °C and 5% CO_2_. Lentiviral transfection was performed using short hairpin RNA (shRNA) sequences, including shTKT: GCTGAGCTGCTGAAGATGTTTTTCAAGAGAAAACATCTTCAGCAGCTCAGC.

### Cell proliferation

Transfected cells and controls were seeded in 96-well plates at a density of 2000 cells/well. Cell proliferation was assessed at 0, 24, 48, and 72 h using the Cell Counting Kit 8 assay, with optical density measured at 450 nm using a microplate reader. Similarly, transfected cells were resuspended and seeded onto crawler plates. After incubation following the instructions of the BeyoClick™ EdU Cell Proliferation Kit with Alexa Fluor 555, 4′,6-diamidino-2-phenylindole (DAPI) staining was conducted to facilitate fluorescence imaging while minimizing exposure to light.

### Colony formation assay

After successful transfection, a total of 3000 cells were cultured in 6-well Petri dishes for 14 days and subsequently stained with crystal violet.

### Western blot assay

Western blotting was performed as described previously. Briefly, cells were lysed on ice for protein concentration determination, denatured at high temperature, and subjected to gel electrophoresis. Proteins were then transferred to membranes, blocked, and incubated with primary antibodies at 4 °C overnight, followed by secondary antibody incubation and visualization. The specific primary antibodies used included CCND1 (Proteintech, 60186-1-Ig), CCND2 (Proteintech, 10934-1-AP), and β-actin (Proteintech, 66009-1-Ig).

### Cell cycle by flow cytometry

Following established protocols, cells were collected, supernatants were discarded, and cells were washed 2–3 times with 2 mL of pre-cooled phosphate-buffered saline solution at 4 °C. Cells were then fixed in pre-cooled 70% alcohol at 4 °C overnight. After incubation with RNaseA (final concentration 200 μg/mL) at 37 °C for 30 min, the enzyme reaction was terminated in an ice bath. Cells were subsequently incubated with 1 mL of propidium iodide (final concentration 20 μg/mL) prepared in phosphate-buffered saline solution at 37 °C for 30 min. Finally, cell cycle analysis was performed using flow cytometry.

### Xenograft tumor model

Approximately 3 million SH-SY5Y cells with stable TKT (transketolase) knockout (shTKT) or negative control (shCtrl) were added to a mixture of 50 mL of medium and 50 mL of matrix gel. Tumor size was monitored using electronic calipers, with the maximum diameter limited to 1 cm. All animal experiments were approved and supervised by the Animal Ethics Committee of CH-CMU.

### Immunohistochemistry assay

Paraffin-embedded sections from NB patients at CH-CMU were subjected to routine dewaxing procedures, followed by antigen retrieval using sodium citrate. Endogenous peroxidase activity was blocked with 3% hydrogen peroxide, before blocking with goat serum, after which the sections were incubated at 4 °C overnight with primary antibody anti-PD-L1 (22C3, DAKO). The following day, after incubation with secondary antibodies and development with DAB (3,3′-diaminobenzidine), the sections were rehydrated and coverslipped.

### Multiplex immunofluorescence assay

CH-CMU-obtained tissue specimens were exposed to Tris-EDTA or citrate buffer antigen retrieval solutions within a microwave-assisted repair cassette. Subsequently, endogenous peroxidase activity was quenched using 3% hydrogen peroxide in a light-shielded environment at room temperature for 25 min. The sections were then blocked with 5% normal goat serum working solution at room temperature for 30 min, followed by overnight incubation with primary antibodies anti-SPP1 (1:100 dilution, Proteintech), anti-IGHM (1:4000 dilution, Proteintech), and anti-CD68 (1:300 dilution, Abcam) in a humidified environment at 4 °C. The following day, sections were treated with secondary antibodies with specific fluorescent labels, nuclei were stained, and the sections were mounted. Imaging was performed thereafter.

### Statistical analysis

Quantitative variables were analyzed using the paired Wilcoxon signed rank test, and student's *t*-test. Overall survival was analyzed using univariate and multivariate Cox proportional risk regression models and Kaplan–Meier curves (log-rank test). *P*-values less than 0.05 were considered statistically significant.

## Results

### Machine learning for predicting NB-BBM occurrence status using histopathological images

**Figure 1:**
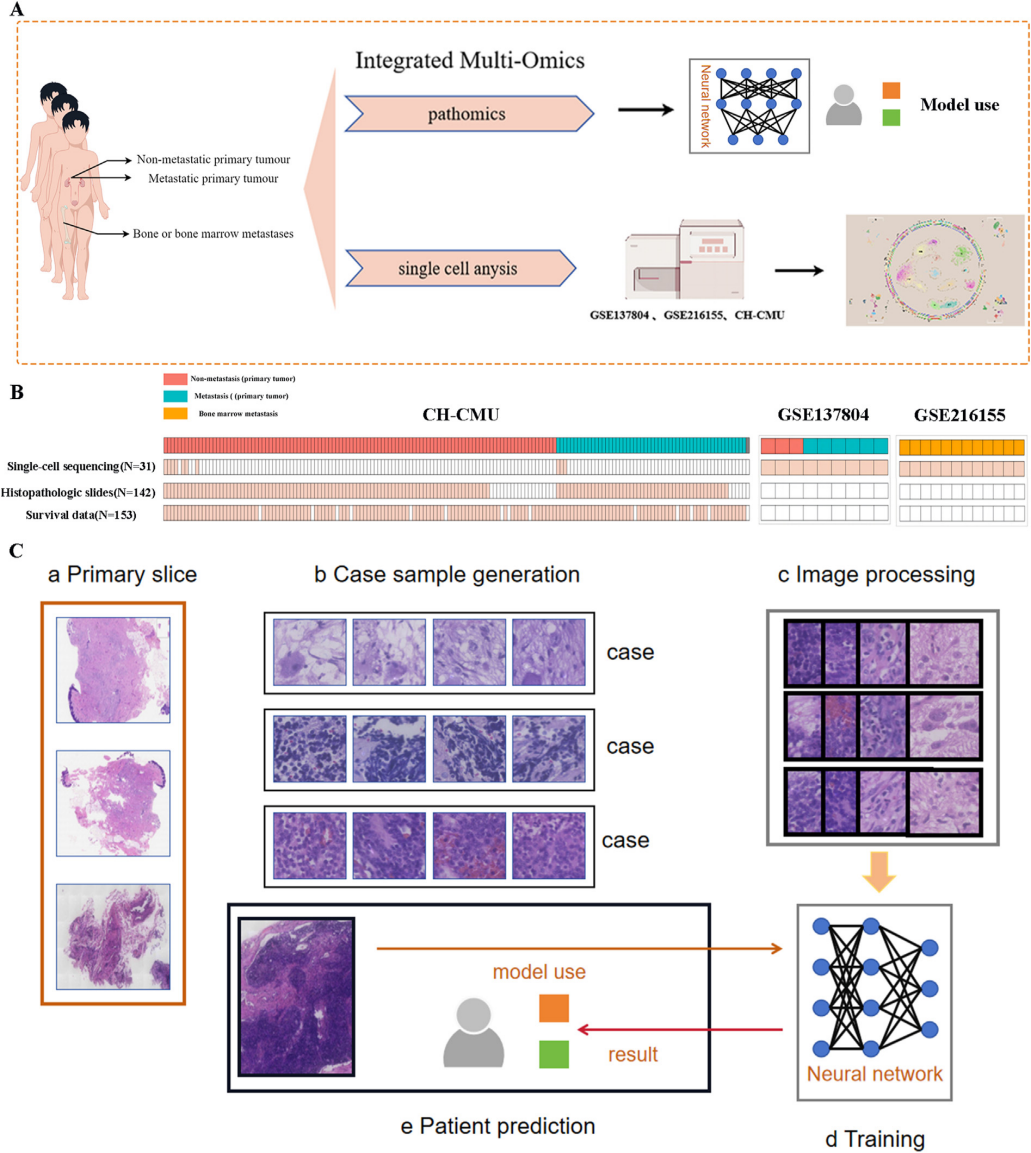
Multi-omics landscape of NB-BBM. **(A)** Experimental scheme for investigating the mechanisms underlying NB-BBM. **(B)** The sample overviews available for single-cell, whole genome sequencing, pathohistological, and survival data. **(C)** Workflow of a deep learning model for a multi-instance learning framework to predict NB-BBM.

A detailed multi-omics study was conducted on a cohort of 167 NB patients from CH-CMU, 142 paraffin-embedded, hematoxylin-eosin-stained tumor sections for NB-BBM prediction, 10 single-cell sequencing datasets, and clinical data for 153 patients (Fig. 1A–C and Table S2).

**Figure 2:**
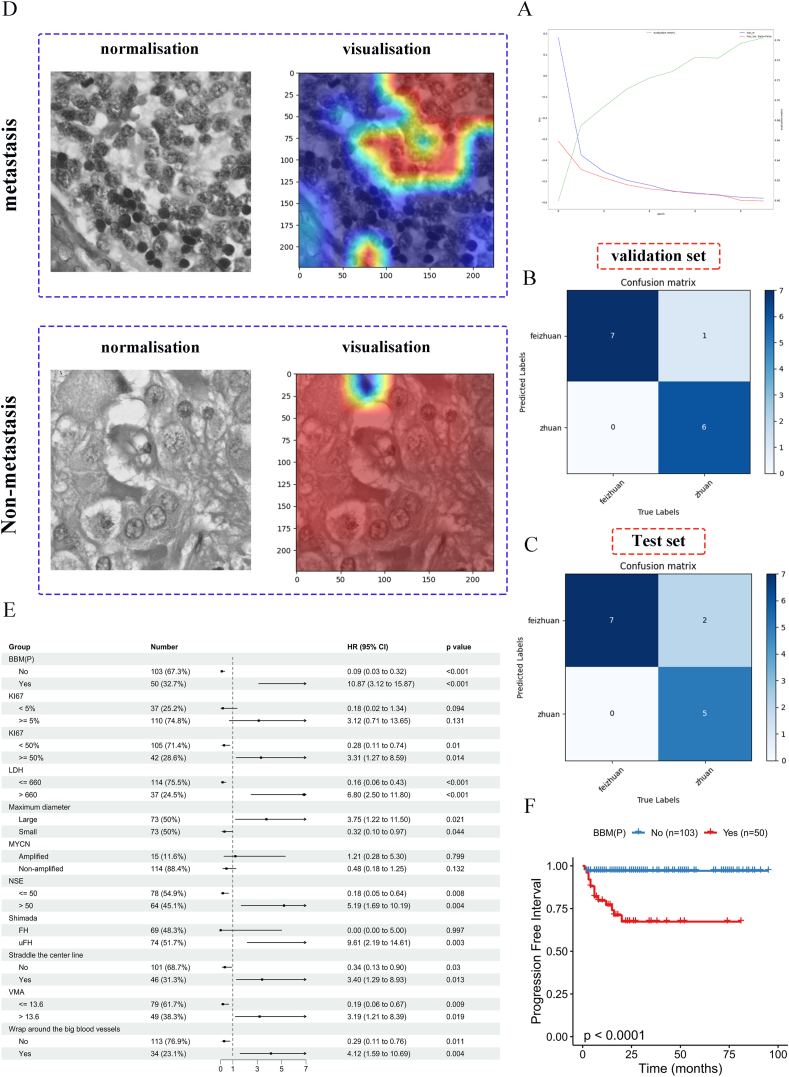
Deep learning models for predicting the performance of NB-BBM and the clinical characteristics of CH-CMU patients. **(A)** Loss values and accuracy changes. **(B, C)** Predictions of the models in the validation and test sets. **(D)** Visualization of NB-BBM with representative specimens of the non-metastatic group. Blue to red indicates an elevated level of model concern. **(E)** The multivariate Cox proportional hazard model applied to the CH-CMU patient cohort. **(F)** The Kaplan–Meier curves for the BBM and non-metastatic groups in the CH-CMU patient cohort.

In this research, tumor areas were marked on pathological images of size 1024 ∗ 1024 pixels by oncology surgeons. The cases were then categorized into the NB-BBM and non-metastatic groups, employing deep multiple Instance Learning for analysis. The pathological images from 142 NB patients were randomly assigned into a training set (114 cases), an independent validation set (14 cases), and a test set (14 cases). After training the model for 100 epochs, an accuracy of 93.14 % was achieved in the training set and 85.7 % in the test set. The progression of loss values and accuracy during training is depicted in Fig. 2A. The model demonstrated reliability in both the validation and test sets, correctly identifying 13 out of 14 cases in the validation set (Fig. 2B, C) and 12 out of 14 cases in the test set. A characteristic feature heatmap was generated to highlight the areas of focus for the classification model, revealing that it distinguished between the NB-BBM and non-metastatic groups based on cellular morphological differences and shape features (Fig. 2D). Additionally, multivariate Cox proportional hazard model analysis of CH-CMU patients with clinical data indicated that those with NB-BBM exhibited higher risk and poorer prognosis (Fig. 2E, F). Thus, an effective pathological image prediction model can significantly aid in diagnosing NB-BBM, facilitating intelligent classification between NB-BBM and non-metastatic groups.

### The single-cell transcriptomic landscape of primary NB and NB-BBM

**Figure 3:**
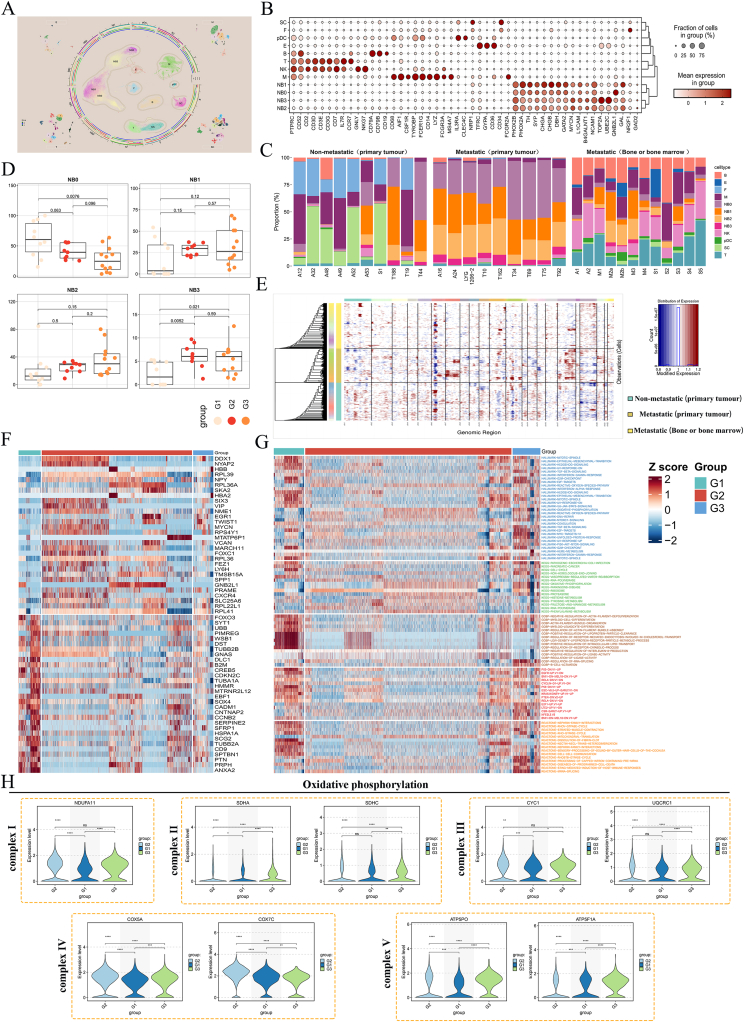
Single-cell landscapes between NB-BBM and non-metastatic groups. **(A)** UMAP of single-cell RNA sequencing data for all cells from 31 neuroblastoma patients. **(B)** Expression of typical cell type marker genes in 12 clusters. **(C)** The percentage of each of the 12 cell subpopulations in the neuroblastoma patient samples. **(D)** Differential profile of each of the 4 tumor cell subpopulations in the G1/G2/G3 groups. **(E)** Tumor cells in the G1/G2/G3 subgroups were analyzed for copy number variations (CNVs). **(F)** Differential genes in NB3 tumor cell subpopulations in the G1/G2/G3 group. **(G)** Pathway enrichment of NB3 tumor cell subpopulations in the G1/G2/G3 group. **(H)** Expression of 5 complex-related molecules in the oxidative phosphorylation pathway in the G1/G2/G3 group.

To characterize cellular heterogeneity and key targets and mechanisms of tumor progression in NB primary tumors and BBM, single-cell transcriptomic analysis was performed on 19 primary NB with BBM and without BBM and 12 BBM samples. Cells from the 31 samples were classified into 12 separate clusters (Fig. 3A, B) based on quality control, data normalization, and principal component analysis.

Initially, we analyzed the composition of primary cell clusters in each sample (Fig. 3C) and found a significantly higher proportion of fibroblasts in non-metastatic NB samples than in those with metastases. This finding, along with pathological staining observations, suggests an association between increased stromal content and less malignant NB subtypes, like the nodular form. Similarly, the distribution of tumor cells in the three subgroups was analyzed, revealing that the NB3 cell subtype was predominantly distributed in the BBM group (Fig. 3D), suggesting this subpopulation of tumor cells may contribute to BBM progression. To further examine the clonal structure of malignant cells in primary NB and BBM groups (G1/G2/G3), the inferCNV algorithm was used to analyze the copy number variations in tumor cells in primary NB and BBM samples. Results revealed that chromosome 3 deletion was more prevalent in the G2/G3 groups than in the G1 group. Moreover, chromosome 7 amplification was more pronounced in the G2 group relative to the G1 group, consistent with earlier findings (Fig. 3E). To further investigate how NB progressed to BBM, we conducted a differential analysis of the NB3 subpopulation of cells in the G1/G2/G3 groups, focusing on pathway enrichment. Results revealed a strong association with the activation of oxidative phosphorylation, suggesting that tumor cells may promote BBM tumorigenesis through metabolic reprogramming (Fig. 3F, G). Subsequently, we compared the expression levels of key genes within the five complexes of oxidative phosphorylation. Consistent with the above results, oxidative phosphorylation was activated when BBM occurred in NB (Fig. 3H).

### NB-BBM-promoting macrophage subpopulations and immune microenvironment

**Figure 4:**
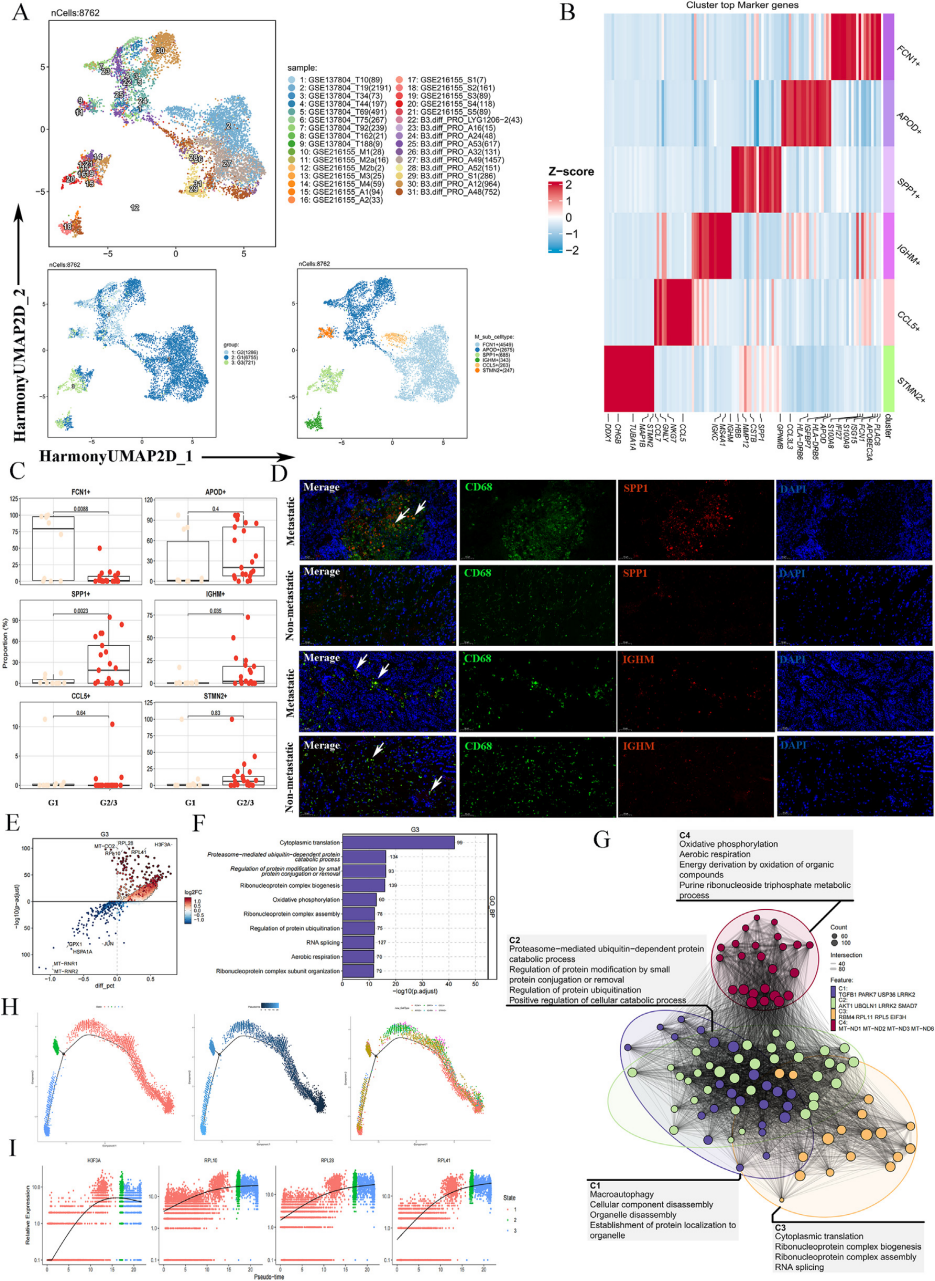
Macrophage subpopulations and immune microenvironment promoting NB-BBM. **(A)** UMAP map of 6 macrophage clusters totaling 8672 cells. **(B)** Heatmap of significantly differentially expressed genes in 6 macrophage clusters. **(C)** Differences in 6 macrophage clusters between the NB-BBM (G2/G3) and non-metastatic (G1) groups. **(D)** Representative immunofluorescence imaging of CD68^+^ SPP1^+^ tumor-associated macrophages (TAMs) and CD68^+^ IGHM^+^ TAMs in human neuroblastoma sections with and without BBM. Arrows indicate CD68^+^ SPP1^+^ TAMs and CD68^+^ IGHM^+^ TAMs. Scale bar, 50 μm. **(E**–**G)** Differential genetic and functional enrichment of the G3 group of the SPP1^+^ TAM subpopulation. **(H, I)** Pseudo-time-ordered analysis of TAMs in neuroblastoma and the expression of genes up-regulated in SPP1^+^ TAMs over pseudo-time.

To explore TAM heterogeneity in NB, TAMs were isolated from 31 NB patients, yielding a total of 8762 TAMs classified into six clusters, annotated as FCN1^+^, APOD^+^, SPP1^+^, IGHM^+^, CCL5^+^, and STMN2^+^ TAMs (Fig. 4A, B). Subsequent analysis of the distribution of the six TAM subtypes between the NB-BBM and non-metastatic groups indicated that the SPP1^+^ TAMs (*P* = 0.0023) and IGHM^+^ TAMs subclusters (*P* = 0.035) were enriched in NB-BBM, suggesting their potential role in promoting NB-BBM (Fig. 4C). Multiplex immunofluorescence assays further verified that CD68^+^ and SPP1^+^ TAMs were significantly enriched in NB-BBM (Fig. 4D).

To clarify the role of the SPP1^+^ TAM subpopulation in NB progression, a detailed analysis was performed on the G3 subgroup of SPP1^+^ TAMs (Fig. 4E). We then explored the phenotypic heterogeneity and dynamic transitions of NB TAMs using Monocle to infer developmental trajectories of cellular lineages. In the G3 group of SPP1^+^ TAMs, the expression of up-regulated genes (H3F3A, PRL10, PRL28, and PRL41) varied with pseudo-time (Fig. 4H, I). Functional enrichment analysis, linking these variations to oxidative phosphorylation and protein ubiquitination regulation, highlighted their importance in tumor BBM, as supported by previous research (Fig. 4F, G).

### Interaction relationship of six TAMs with tumor cells

Analysis of the potential interactions between NB and other macrophage subpopulations, especially the SPP1^+^ TAM subpopulation, revealed close communication between the NB3 subpopulation and related macrophage subpopulations (Fig. S2A). Therefore, we focused on the activation of cellular pathways in the G3 group, particularly the SPP1^+^ TAM subpopulation and pathways associated with NB3, such as ADGRE5, ANNEXIN, CD99, CHD, GRN, MK, PTN, and TGFβ signaling (Fig. S2B). Subsequently, we analyzed the contribution of marker gene interactions of the SPP1^+^ TAM subpopulation-associated signaling pathways to intercellular subpopulation communication. Results indicated that CD99-CD99 interactions played a significant role in facilitating communication between SPP1^+^ TAMs and NB3 cells, particularly in the SPP1^+^ TAM-NB2 subpopulation (Fig. S2C).

### Potential immunotherapeutic targets in NB-BBM

**Figure 5:**
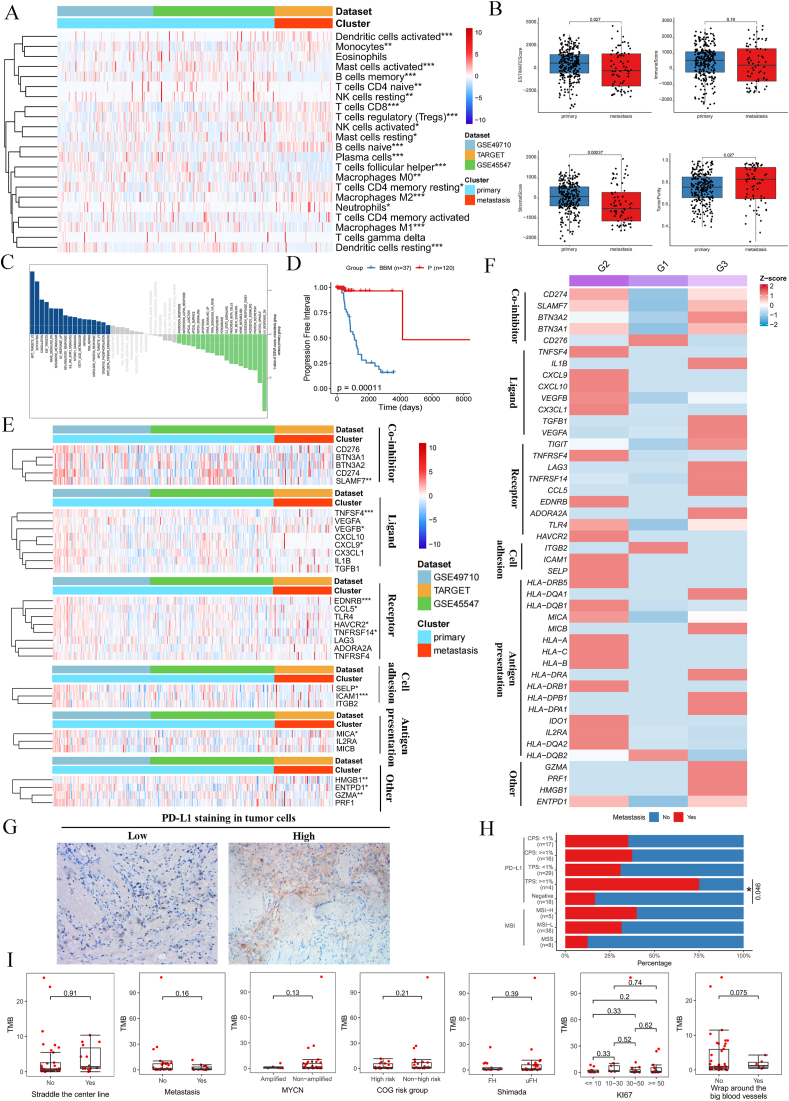
Comparison of immune infiltration and immune targets in NB-BBM and non-metastatic groups. **(A)** Microenvironmental phenotypic landscape of NB-BBM and the heatmap showing the estimated number of 22 microenvironmental cell subpopulations based on CIBERSORT calculations (primary, blue; metastatic, red). **(B)** Scores of stromal, immune, tumor purity, and ESTIMATE between NB-BBM and non-metastatic groups. **(C)** Gene set variation analysis (GSVA) shows the differential pathways in the NB-BBM and non-metastatic group samples. **(D)** Prognostic and survival analysis of the NB-BBM and non-metastatic groups. **(E, F)** Heatmap of immune-related targets between the NB-BBM and non-metastatic groups in transcriptomic and single-cell data. **(G, H)** Representative images (left panel) and quantitative results (right panel) of PD-L1 immunohistochemical staining in 51 neuroblastoma samples. Quantitative representation of PDL1 staining and microsatellite instability (MSI) in NB-BBM and non-metastatic groups. **(I)** Tumor mutational burden (TMB) between the NB-BBM and non-metastatic groups in 51 neuroblastoma patients in CH-CMU.

Despite the limited effectiveness of immunotherapy, particularly immune checkpoint inhibitors, in many pediatric solid tumors, this study aimed to identify viable immunotherapeutic targets for NB-BBM, paving the way for the development of antibody-based treatments such as naked antibodies, antibody–drug conjugates, and bispecific antibodies. Initially, we focused on immune-cell infiltration within the NB-BBM subset, using data from the GSE49710, GSE45547, and TARGET datasets, and identified a notable decrease in M1 macrophage infiltration and increase in M2 macrophage infiltration in the NB-BBM group (Fig. 5A). The estimation algorithm indicated that the NB-BBM group exhibited a low stromal score and high tumor purity (Fig. 5B). Furthermore, GSVA highlighted significant activation of pathways related to MYC targets, glycolysis, IL6/JAK/STAT3, and oxidative phosphorylation in the BBM group (Fig. 5C). The Kaplan–Meier survival curves also indicated that the BBM group exhibited poor prognosis (Fig. 5D).

To identify potential immune targets in NB-BBM patients and examine their role in tumor immune infiltration, a comparative analysis of gene expression between metastatic and non-metastatic tumors was conducted (Fig. 5E, F). Results revealed that CD274, LAG3, and TIGIT were significantly up-regulated in the G3 (BM) group, based on single-cell transcriptome data. Immunohistochemical analysis of PD-L1 in a cohort of 51 NB patients further demonstrated pronounced expression in the NB-BBM group, with a tumor proportion score (PD-L1 protein-expressing tumor cells as a proportion of whole tumor cells) > 1 distinguishing metastatic and non-metastatic NB patients (*P* = 0.046) (Fig. 5G, H).

Additionally, we investigated microsatellite instability and tumor mutational burden as predictors of immunotherapy efficacy between the NB-BBM and non-metastatic groups. Previous studies have suggested that microsatellite instability is uncommon in NB, with the tumor mutational load categorizing primary NB with the lowest mutation rates among cancers, corresponding with the “immunocold” phenotype. Consistently, our findings revealed no significant disparities in microsatellite instability or tumor mutational burden between the NB-BBM and non-metastatic groups, indicating the need for alternative strategies to effectively target NB (Fig. 5I).

### Integrated transcriptomics and single-cell analyses confirm metabolism-related molecule TKT as a potential regulator of NB-BBM

**Figure 6:**
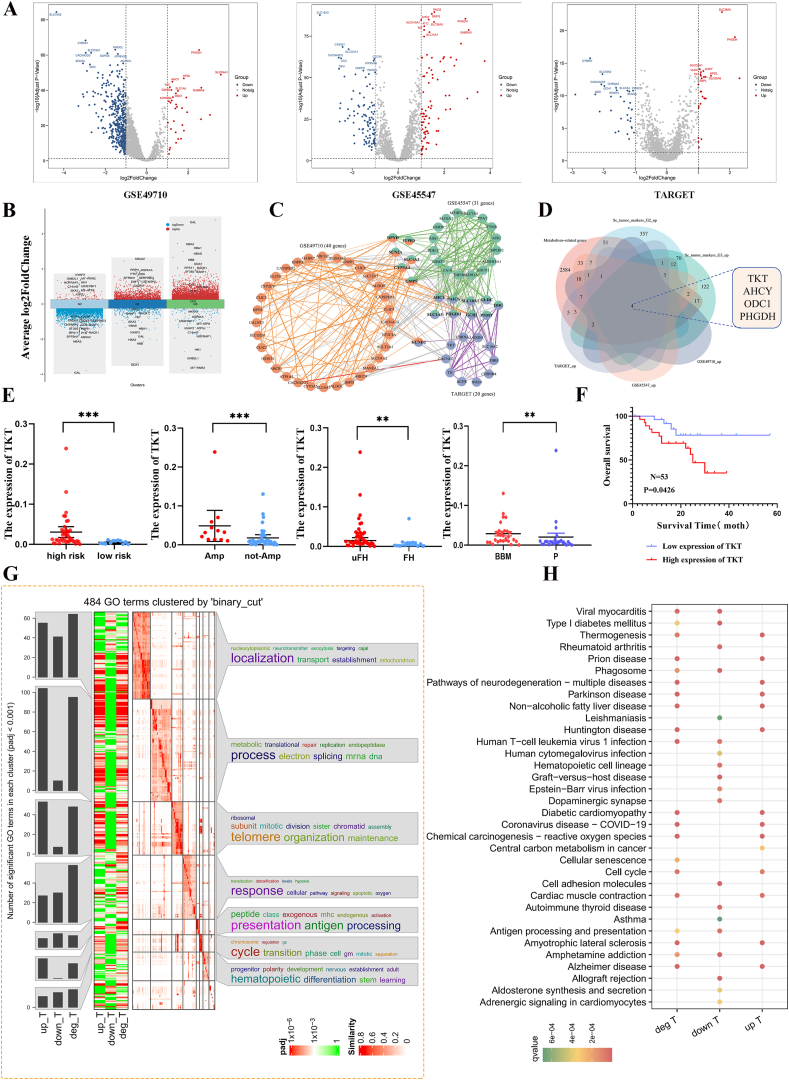
Integrated transcriptomic and single-cell analyses identified TKT as a potential regulatory molecule for NB-BBM. **(A)** Differential genes between MYCN amplified and non-amplified intergroups in the GSE49710, GSE45547, and TARGET datasets. **(B)** Up- and down-regulated genes between the G1/G2/G3 groups of the single-cell dataset. **(C)** Core genes were identified using Cytoscape in the GSE49710, GSE45547, and TARGET datasets. **(D)** Key metabolic genes in the up-regulated genes of the G2/G3 group and the up-regulated genes in the three neuroblastoma transcriptome cohorts in the single-cell data. **(E)** Relative expression of TKT in neuroblastoma patients with or without high risk, MYCN amplification, unfavorable histology (uFH), and BBM. **(F)** Survival curves were plotted by dividing neuroblastoma patients into high- and low-expression groups based on TKT expression. **(G, H)** Gene Ontology (GO) and Kyoto Encyclopedia of Genes and Genome (KEGG) enrichment analyses were performed in the GSE49710, GSE45547, and TARGET merged datasets, which were divided into high- and low-expression groups based on the median TKT mRNA expression.

To identify key drivers of BBM development in NB, we initially analyzed single-cell data from up and down-regulated genes across the G1/G2/G3 groups (Fig. 6B), then performed transcriptome analysis of the GSE49710, GSE45547, and TARGET datasets. Given the close association between MYCN amplification and NB-BBM, we divided each of the three transcriptome cohorts into MYCN-amplified and non-amplified groups for differential analysis (Fig. 6A). Cytoscape was then used to identify core genes among the differentially expressed genes in the three NB cohorts (Fig. 6C). Based on analysis of the up-regulated genes in the G2/G3 group within the single-cell dataset and in the three NB transcriptome cohorts, four key targets related to metabolism were identified: TKT, AHCY, ODC1, and PHGDH (Fig. 6D). The distribution and expression of these four key targets in single cells and the G1/G2/G3 groups were further determined (Fig. S3B, C). We focused on the metabolism-related molecule TKT, whose mechanism has not been studied in NB. Analysis of TKT expression in the 53 CH-CMU-obtained NB tumor tissues indicated TKT enrichment in BBM (Fig. 6E and Table S3). Furthermore, survival analysis indicated that a higher level of TKT was associated with poorer prognosis in NB patients (Fig. 6F; Fig. S3D).

**Figure 7:**
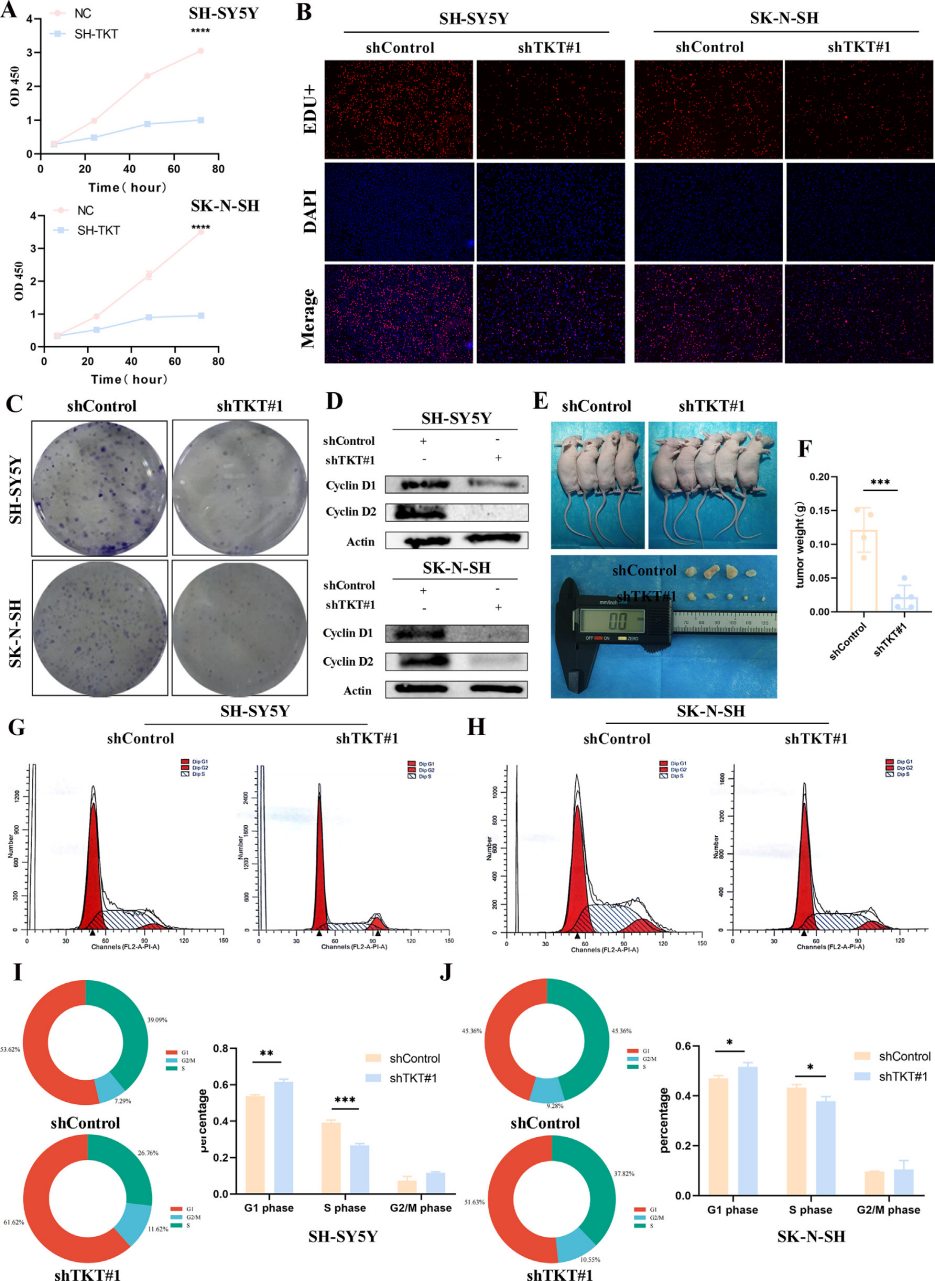
TKT controls neuroblastoma cell growth *in vitro* and *in vivo*. **(A)** The growth rate of neuroblastoma cells was significantly reduced after TKT knockdown as detected by the CCK-8 assay. **(B)** 5-Ethyl-2-deoxyuridine (EdU) assay showed that down-regulation of TKT reduced the growth of neuroblastoma cells. **(C)** Colony formation assays showed that in neuroblastoma cells, knockdown of TKT in neuroblastoma cells reduced the size of colony formation. **(D)** Effects on CCND1 and CCND2 protein expression after knockdown of TKT in neuroblastoma cells. **(E, F)** Subcutaneous tumor formation. TKT knockdown resulted in a reduction in tumor size and weight versus control. **(G**–**J)** Effect of TKT on cell cycle progression in neuroblastoma cells detected by flow cytometry.

To explore the potential molecular mechanisms underlying TKT regulating NB progression and BBM occurrence, Gene Ontology (GO) and KEGG pathway analyses were performed on differentially expressed genes between groups with high and low TKT expression. Results revealed that TKT up-regulation was closely related to cell cycle processes (Fig. 6G, H). We selected two cell lines, SK-N-SH and SH-SY5Y, to explore the potential function of TKT in NB cells. Stable transfected cells were successfully constructed using lentivirus. Quantitatively, cell growth was significantly reduced after TKT knockdown (Fig. 7A). Measured with EdU, the results showed the same trend of both cell lines, with reduced proliferation in the knockdown group (Fig. 7B). Colony formation assay showed that cell growth was reduced in the TKT knockdown group in both cell lines compared with the control group (Fig. 7C). The knockdown of TKT significantly reduced the protein expression of CCND1 and CCND2 in the cells (Fig. 7D), suggesting that TKT may affect the mitotic G1/S phase of NB cells. We also used flow cytometry to detect the effect of TKT on the cell cycle process. The results showed that TKT knockdown delayed the cellular G1–S phase transition, and the number of SH-SY5Y cells lagging to the G1 phase increased from 53.62% to 61.62%, and the S phase decreased from 39.09% to 26.76%. Similarly, the same trend was obtained for another cell line of NB (Fig. 7G–J). Finally, stably transfected TKT and control SH-SY5Y cells were injected subcutaneously into the skin of nude mice. The results showed that TKT knockdown significantly reduced the tumor volume and weight compared with the control (Fig. 7E, F).

## Discussion

This study offers an in-depth analysis of the use of artificial intelligence for pathological diagnosis in NB, particularly for predicting BBM transformation, alongside the genomic and single-cell transcriptomic alterations found in NB-BBM. Our findings underscore the importance of predictive pathological diagnosis for NB-BBM, as well as exploring the onset and progression of NB and the tumor heterogeneity observed in distant BBM.

Leveraging deep learning to analyze histological images presents significant potential for identifying patterns associated with tumor characteristics, enabling the prediction of aggressive biological behaviors such as the propensity for metastasis.^19^ Previous deep-learning models based on NB pathological images have focused on evaluating tumor differentiation and providing prognostic assessments.^20^, ^21^, ^22^ To the best of our knowledge, this study marks the first application of deep learning models to pathological images specifically for predicting BBM in NB. Notably, our predictive model, based on pathology slide images and the Swin-Transformer algorithm, achieved a classification accuracy exceeding 85%, thus contributing to more precise imaging evaluations and supporting timely intervention for NB patients at heightened risk of BBM.

Cancer metabolism has regained widespread research interest in recent years.^23^ The focus has been mainly on the primary tumor, while metabolic regulation during dissemination has been less well-studied. In our single-cell study, we focused on alterations in metabolism during progression from the primary lesion to tumor progression to bone or bone metastasis, and we found that oxidative phosphorylation was emphatically involved in the development of BBM in patients with NB, where the key molecules in complexes I–V were significantly higher expressed in the metastatic group. It has been reported that some cancer cells, especially with high metastasis, are more dependent on OXPHOS.^24^ The absence of a retinoblastoma (RB1) tumor suppressor in breast cancer induces OXPHOS, which plays a central role in promoting metastasis.^25^ Meanwhile, in NB, DLST deficiency significantly inhibits NADH production and impairs OXPHOS, leading to growth arrest and apoptosis of NB cells. In addition, multiple inhibitors targeting the electron transport chain, including potent IACS-010759, which is currently undergoing clinical trials in other cancers, effectively reduced NB proliferation *in vitro*.^26^ All these results reveal that OXPHOS is an important factor in the progression of NB metastasis.

Remarkably, the TKT gene was identified through single-cell transcriptomics as a crucial metabolic molecule linked to BBM. Previous studies have confirmed its role in promoting tumor proliferation and metastasis.^27^^,^^28^ Consistently, our findings showed that the TKT gene was strongly associated with the clinical features of NB patients, especially in the BBM group. Subsequent *in vivo* and *in vitro* experiments even further validated the malignant biological behavior of TKT. Pathway enrichment analysis revealed that high TKT expression was correlated with cell cycle activity, while NB cell lines demonstrated that down-regulation of the TKT gene led to a decrease in CCND1, CCND2, and other cell cycle-related proteins.

Our results indicated that immunosuppression played a key role in the NB-BBM group. At the genomic level, no significant differences in tumor mutational load were observed between the BBM and non-metastatic groups, consistent with existing studies.^29^^,^^30^ NB employs a variety of immune evasion strategies, including aberrant expression of immune checkpoint molecules.^31^ Tumor cells are known to hijack the PD-1/PD-L1 pathway to evade immune surveillance.^32^^,^^33^ Elevated PD-L1 expression was observed in the BBM group based on single-cell analysis, with protein expression differences noted between the BBM and non-metastatic groups in the CH-CMU cohort. Furthermore, NB induces immunosuppressive myeloid cells that secrete immunomodulatory mediators, thereby affecting immune cell infiltration and function. TAMs, which constitute a significant portion of the immune cell population in tumors, are critical for NB prognosis.^34^^,^^35^ They are known to secrete TGF-b1,^36^ which directly targets cytotoxic T cells and participates in the differentiation of regulatory T cells.^37^, ^38^, ^39^ Our research also highlighted the enrichment of SPP1^+^ TAMs in NB-BBM, which closely interacted with the metastatic subpopulation of NB bone marrow (NB3) and potentially contributed to NB progression to BBM through oxidative phosphorylation and aberrant TGF-b1 expression. However, further experiments are needed to provide conclusive evidence on the role of SPP1^+^ TAMs in NB-BBM.

In conclusion, our work offers a pathodiagnostic prediction for the risk of NB-BBM, enhances other imaging diagnoses, and elucidates the cellular heterogeneity of initial, progressive, and distant metastatic sites in NB. However, our study has several limitations. First, there is a need for multicenter validation of the model for predicting NB-BBM status, and given the retrospective nature of this study, prospective research is required to confirm its clinical utility. Second, despite the multi-omics approach, additional experiments are necessary to verify the reliability and usefulness of the identified targets. Future strategies should explore linking critical molecules with targeted drugs through nuclides, offering a novel avenue to improve NB-BBM prognosis.

## Ethics declaration

The Ethics Committee of Children's Hospital Affiliated to Chongqing Medical University approved the use of retrospective patient material (ethics number: 2023-534). Written informed consent was obtained from all participants.

## Funding

This work was supported in part by research grants from the Key Project of the National Key R&D Plan “Research on Prevention and Control of Major Chronic Non-Communicable Diseases” (China), the Ministry of Science and Technology of the People's Republic of China, and the 10.13039/501100012166National Key R&D Program of China (No. 2018YFC1313000, 2018YFC1313004).

## CRediT authorship contribution statement

**Kai Huang:** Data curation, Formal analysis, Software, Writing – original draft. **Linyu Yang:** Methodology, Writing – review & editing. **Yue Ma:** Writing – review & editing. **Lijian Cao:** Writing – review & editing. **Suwen Li:** Writing – review & editing. **Zhenzhen Zhao:** Writing – review & editing. **Jianwu Zhou:** Writing – review & editing. **Shan Wang:** Conceptualization, Funding acquisition, Writing – review & editing.

## Data availability

The public data used in this work are available from the Gene Expression Omnibus database (GEO, http://www.ncbi.nlm.nih.gov/geo/). Single-cell data from NB patients at CH-CMU, as well as original experimental data and analysis code supporting the conclusions of this paper, will be provided by the corresponding author.

## Conflict of interests

There were no competing interests to disclose.

## Figures and Tables

**Figure:**
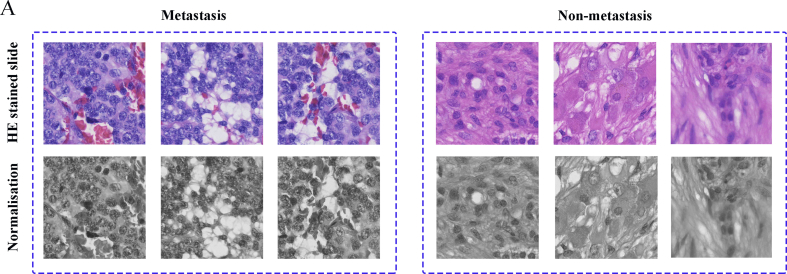
Figure S1 Pathological images were converted to greyscale by the OpenCV library.

**Figure:**
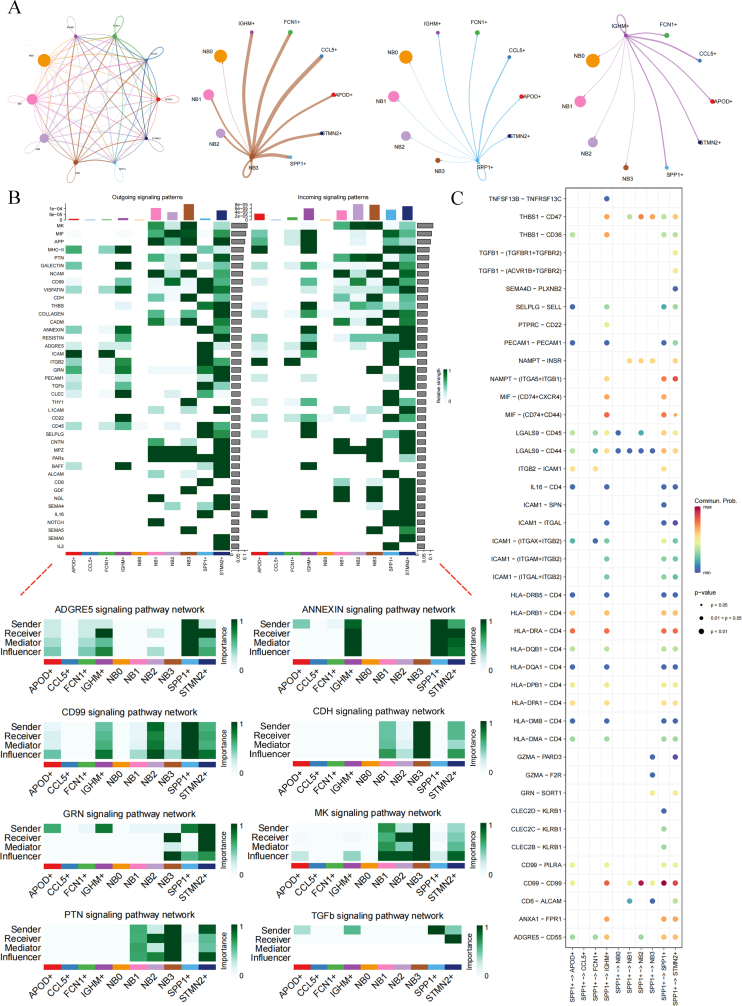
Figure S2 Interaction of six tumor-associated macrophage (TAM) subclusters with tumor cells. **(A)** Communication links between ten single-cell subtypes. Weights of interactions between SPP1^+^, NB3, and IGHM^+^ cell subpopulations and other subclasses of cell populations. **(B)** Input and output signaling pathways for each cell type. Signaling pathways associated with SPP1^+^ TAMs and NB3 cell subpopulations are highlighted. **(C)** Significantly correlated ligand-receptor interactions between subpopulations of SPP1^+^ TAM cells and most communicating cell types.

**Figure:**
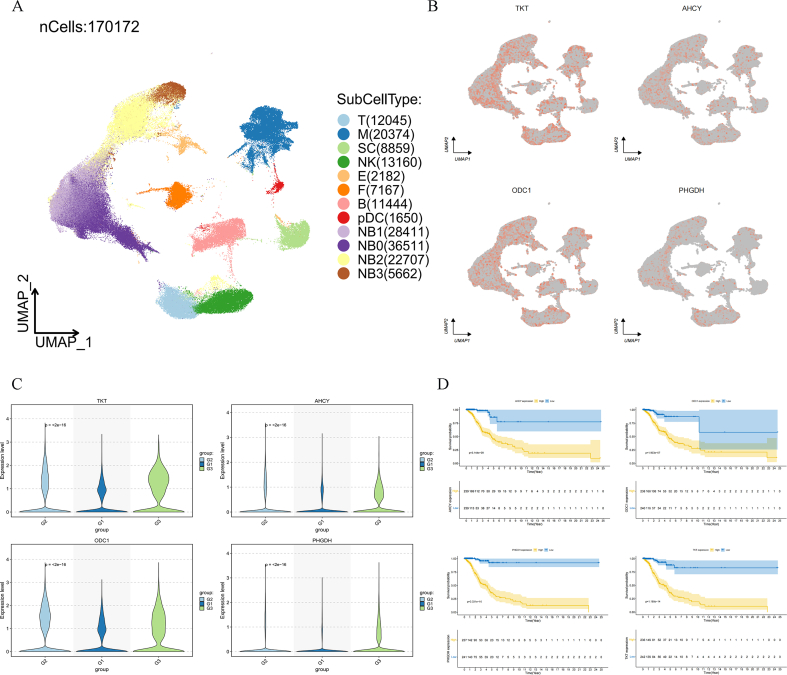
Figure S3 Distribution of TKT, ODC1, AHCY, and PHGDH in single cells and their survival curves. **(A)** Distribution of 12 subpopulations of cells in single-cell data. **(B, C)** Distribution of TKT, AHCY, ODC1, and PHGDH in single-cell data and their expression in the G1/G2/G3 groups. **(D)** Survival curve analysis of TKT, AHCY, ODC1, and PHGDH in the GSE49710 dataset.
