# Supplementary material for: Integrated multi-omics characterization of neuroblastoma with bone or bone marrow metastasis
Source: Genes Dis. 2025 Jan 3;12(3):101511. doi: 10.1016/j.gendis.2024.101511 (PMC11894310; doi:10.1016/j.gendis.2024.101511)
Supplement: Multimedia component 1 [file mmc1.docx]

**Supplementary materials and methods**

**Classification of pathology images using Multiple Instance Learning**

The Multiple Instance Learning (MIL) paradigm is a type of supervised learning that differs from the conventional single sample approach by incorporating training data in the form of a 'bag'. In the context of medical imaging, a medical image can encompass many regions, with only a subset of these regions exhibiting lesions. By employing multi-sample learning, it is possible to identify the affected area within the entire image. In this study, the retrieved picture characteristics are classified using the utilization of MIL.In this study, features from 400 images were extracted for each case, and these 400 feature tensors formed a case package that was used as a sample for multi-instance classification.

**Tumor mutational burden (TMB) and microsatellite instability (MSI)**

Using high-throughput sequencing, we analyzed the length distribution of 27 microsatellite loci, including BAT-25, BAT-26, D2S123, D5S346, and D17S250, in 50 NB patients（CH-CMU）.Subsequently, we analysed the differences in TMB or MSI between patients with BBM and those with primary NB.

**Bulk RNA-seq analysis**

RNA-seq data were downloaded from the GEO database（ GSE49710, GSE45547, and TARGET datasets）. The statistical analysis of NB patients, distinguishing between NB-BBM and primary groups, was executed using R, specifically employing the ggplot2 and limma packages to identify immune-related molecules and illustrate the findings. Results yielding p-values below 0.05 were deemed statistically significant.

**Survival analysis**

Survival outcomes were assessed between BBM and primary cases across various datasets. Kaplan-Meier (K-M) curves were constructed to visualize survival differences, with the log-rank test applied to ascertain the significance of the observed differences. Additionally, the Cox proportional hazards model was applied to calculate the hazard ratios (HR) and their 95% confidence intervals (CI), utilizing the “survival” package in R. Graphical representations included a horizontal line denoting the 95% CI for each factor analyzed and a vertical dashed line indicating an HR of 1, with HR values greater than 1 correlated with poorer prognosis.Subsequently, the GSE49710, GSE45547, and TARGET datasets were consolidated, normalized, and categorized into high and low expression groups based on TKT expression levels for further Gene Ontology (GO) and KEGG enrichment analyses.

**Estimation of immune cell types**

The normalized expression matrices from the GSE49710 and GSE45547 NB datasets, combined with the TARGET project data, were analyzed using “CIBERSORT R script v1.03” to measure the abundance of 22 immune cell subpopulations within each sample. This enabled a comparative analysis of immune cell infiltration between the BBM and P groups within the cohort. The “estimate” package in R was then used to calculate StromalScore, ImmuneScore, ESTIMATEScore, and TumorPurity metrics for the BBM and P cohorts, facilitating assessment of differences in immune regulation between the two cohorts and identifying key immunoregulatory factors between these groups.

**Table S1.**The Primers used in quantitative real-time PCR (qPCR)

| **Name** | **Sequence (5’-3’)** |
| --- | --- |
| TKT_primer_F | CGCCAATACAAAGGGTATCTG |
| TKT_primer_R | TTTCTTTCTTCAGCAGTTCGG |

**Table S3.** Clinicopathological characteristics of patient samples and expression of TKT in Neuroblastoma .

| Characteristics | Low expression of TKT | High expression of TKT | P value |
| --- | --- | --- | --- |
| n | 26 | 27 |  |
| Age（18 month）, n (%) |  |  | 0.215 |
| ≥18 | 22 (41.5%) | 19 (35.8%) |  |
| ＜18 | 4 (7.5%) | 8 (15.1%) |  |
| Gender, n (%) |  |  | 0.685 |
| female | 13 (24.5%) | 12 (22.6%) |  |
| male | 13 (24.5%) | 15 (28.3%) |  |
| COG risk, n (%) |  |  | < 0.001 |
| low risk | 11 (20.8%) | 1 (1.9%) |  |
| high risk | 15 (28.3%) | 26 (49.1%) |  |
| uFH, n (%) |  |  | 0.003 |
| FH | 11 (20.8%) | 2 (3.8%) |  |
| uFH | 15 (28.3%) | 25 (47.2%) |  |
| MYCN, n (%) |  |  | < 0.001 |
| Not_Amp | 26 (49.1%) | 15 (28.3%) |  |
| Amp | 0 (0%) | 12 (22.6%) |  |
| Bone_marrow_metastasis, n (%) |  |  | 0.002 |
| No | 19 (35.8%) | 8 (15.1%) |  |
| Yes | 7 (13.2%) | 19 (35.8%) |  |
| Bone_metastasis, n (%) |  |  | 0.324 |
| No | 23 (43.4%) | 20 (37.7%) |  |
| Yes | 3 (5.7%) | 7 (13.2%) |  |
| Bone_marrow_or_bone_metastasis, n (%) |  |  | < 0.001 |
| No | 18 (34%) | 6 (11.3%) |  |
| Yes | 8 (15.1%) | 21 (39.6%) |  |
